# Global and Regional Differences in Brain Anatomy of Young Children Born Small for Gestational Age

**DOI:** 10.1371/journal.pone.0024116

**Published:** 2011-09-13

**Authors:** Henrica M. A. De Bie, Kim J. Oostrom, Maria Boersma, Dick J. Veltman, Frederik Barkhof, Henriette A. Delemarre-van de Waal, Martijn P. van den Heuvel

**Affiliations:** 1 Department of Pediatrics, Vrije Universiteit Medical Center, Amsterdam, The Netherlands; 2 Neuroscience Campus Amsterdam, Vrije Universiteit University Medical Center, Amsterdam, The Netherlands; 3 Department of Pediatric Psychology, Vrije Universiteit University Medical Center, Amsterdam, The Netherlands; 4 Department of Clinical Neurophysiology, Vrije Universiteit University Medical Center, Amsterdam, The Netherlands; 5 Department of Psychiatry, Vrije Universiteit University Medical Center, Amsterdam, The Netherlands; 6 Department of Radiology, Vrije Universiteit University Medical Center, Amsterdam, The Netherlands; 7 Department of Pediatrics, Leiden University Medical Center, Leiden, The Netherlands; 8 Neuroimaging Division, Rudolf Magnus Institute of Neuroscience, University Medical Center Utrecht, Utrecht, The Netherlands; Hôpital Robert Debré, France

## Abstract

In children who are born small for gestational age (SGA), an adverse intrauterine environment has led to underdevelopment of both the body and the brain. The delay in body growth is (partially) restored during the first two years in a majority of these children. In addition to a negative influence on these physical parameters, decreased levels of intelligence and cognitive impairments have been described in children born SGA. In this study, we used magnetic resonance imaging to examine brain anatomy in 4- to 7-year-old SGA children with and without complete bodily catch-up growth and compared them to healthy children born appropriate for gestational age. Our findings demonstrate that these children strongly differ on brain organisation when compared with healthy controls relating to both global and regional anatomical differences. Children born SGA displayed reduced cerebral and cerebellar grey and white matter volumes, smaller volumes of subcortical structures and reduced cortical surface area. Regional differences in prefrontal cortical thickness suggest a different development of the cerebral cortex. SGA children with bodily catch-up growth constitute an intermediate between those children without catch-up growth and healthy controls. Therefore, bodily catch-up growth in children born SGA does not implicate full catch-up growth of the brain.

## Introduction

An optimal intrauterine environment is vital for normal brain development. The development of the neural system starts in the third week of gestation and progresses throughout pregnancy and after birth [1]. Postnatal environmental factors and genetic influences contribute to eventual outcome [2]–[4]. Adverse circumstances such as placental insufficiency can interfere with brain development [5]–[10]. The effects of intrauterine disturbances on brain development can extend into adulthood [10]–[12].

In children who are born small for gestational age (SGA), a suboptimal intrauterine environment has lead to underdevelopment of both the body and the brain [13]–[15]. Intrauterine growth restriction is most commonly caused by placental insufficiency [16]. SGA is characterized by decreased body length and/or weight and a diminished head circumference at birth. In the majority of these children, the delay in body growth is spontaneously restored during the first two years of life (SGA+) [13]. Approximately 10% lack catch-up growth and exhibit persistent short stature (SGA−). In addition to a negative influence on physical parameters, decreased intelligence levels and impaired cognitive function have been described in SGA children [17], [18]. This is exemplified by SGA children having a poorer school performance and experiencing more learning difficulties compared to healthy children [19], [20]. Interestingly, catch-up growth of body and/or head circumference is associated with relatively better cognitive outcome [9], [17], [21].

There is, however, limited knowledge on how being born SGA affects human brain anatomy. A cohort of prematurely born infants born SGA displayed lower total brain volume with lower cerebral cortical grey matter volume compared to premature infants born appropriate for gestational age (AGA) [14]. Another cohort of 15 years old SGA adolescents demonstrated lower total brain volume with reduced white matter volume but no significant differences in grey matter volume compared to healthy controls [11], [12], but only children with postnatal catch-up growth were included. It remains unknown, therefore, to what extent the development of the brain parallels the catch-up growth of the body in SGA children.

In the current study we performed a magnetic resonance imaging (MRI) study to investigate whether, and if so, how brain anatomy is affected in young children born small for gestational age and whether bodily catch-up growth parallels catch-up in brain anatomy. To this aim, we examined differences in both global volumes of cerebral and cerebellar structures as well as regional changes in cortical integrity in 4–7 year old SGA children. Regional analysis was performed using parcellation of the cortical mantle, exploring focal differences in thickness of the cortical mantle. The effect of catch-up growth on brain anatomy was studied by comparing a group of SGA children who had recently completed their bodily catch-up growth to a group of SGA children with persistent short stature. In addition, relationships between brain anatomy and IQ were investigated.

## Materials and Methods

### Participants

The present study is part of a longitudinal study on brain development and cognition in children born small for gestational age (SGA) (Dutch Trial Register: NTR 865). The study cohort consisted of 55 children. Children were between 4 and 7 years old at the time of the study. Nineteen children were born AGA and 36 were born SGA. Of these 36 SGA children, 21 displayed postnatal catch-up growth (SGA+) and 15 children had persistent short stature (SGA−) (Table 1). Following the International Small for Gestational Advisory Consensus Board Development Conference Statement (2003), SGA was defined as a birth weight and/or birth length ≤−2SD, adjusted for gender and gestational age; SGA+ was defined as postnatal catch-up growth with an actual height of less than 2 SD below the mean; and SGA− as persistent postnatal growth failure based on an actual height of less than 2.5 SD below the mean [22]. SGA children were selected from the pediatric departments of the VU University Medical Center or one of the other participating hospitals in The Netherlands. Exclusion criteria were 1) severe prematurity below 34 weeks, 2) multiple birth, 3) complicated neonatal period with signs of severe asphyxia, defined as an Apgar score <7 after 5 min, 4) growth failure caused by other somatic or chromosomal disorders or syndromes (except for Silver-Russell syndrome), 5) previous or present use of medication that could interfere with growth or GH treatment and 6) severe learning disability (IQ<70). For optimal comparison, the group of AGA children was matched for age, gender and gestational age with the SGA group.

**Table 1:** Characteristics of study groups (n = 55 children).

					Main effect subgroup analysis (AGAvsSGA+vsSGA−)	
	AGA (N = 19)	SGA				
		SGA+ (N = 21)	SGA− (N = 15)	SGA total group (N = 36)	F value	p value
Gender (boys∶girls)	10∶9	11∶10	9∶6	20∶16		ns
Handedness (right∶left)	17∶2	18∶3	13∶2	31∶5		ns
Gestational age in weeks	39.5 (1.8)	38.8 (1.9)	39.2 (2.0)	38.9±1.9	0.7	ns
Birth weight in grams	3518 (604)	2200 (354)	2458 (467)	2308 (419)	40.3	<0.0001
Birth weight SD	0.4 (0.9)	−2.6 (0.4)	−2.4 (0.4)	−2.5 (0.4)	129.3	<0.0001
Head circumference SD at birth	0.0 (0.7)	−1.1 (0.6)	−0.9 (1.0)	−1.1 (0.8)	8.9	0.001
Age at MRI investigation in years	5.9 (1.0)	5.9 (1.0)	5.7 (1.0)	5.8(0.9)	0.3	ns
Length at MRI investigation in cm	118.6 (9.0)	116.9 (6.6)	102.7 (6.0)	111.0 (9.5)	22.8	<0.0001
Length SD at MRI investigation	0.0 (0.9)	−0.4 (0.8)	−3.0 (0.3)	−1.5 (1.5)	80.7	<0.0001
Weight at MRI investigation in kilograms	22.5 (6.1)	20.3 (3.7)	14.8 (1.6)	17.9 (4.1))	13.5	<0.0001
Weight SD at MRI investigation*	−0.1 (0.7)	−0.6 (1.1)	−1.4 (0.9)	−1.0 (1.0)	7.4	0.002
Head circumference at MRI investigation in cm	52.1 (1.6)	51.3 (1.1)	49.1 (1.7)	50.5 (1.7)	17.5	<0.0001
Head circumference SD at MRI investigation	0.5 (0.7)	0.0 (0.6)	−1.3 (1.0)	−0.5 (1.0)	22.0	<0.0001

Data (except gender and handedness) are presented as mean (± standard deviation); p-value<0.05 is considered significant, p-values between 0.05 and 0.10 are reported.

*: weight for length SD.

Abbreviations: AGA: appropriate for gestational age; SGA+ small for gestational age with postnatal catch up growth; SGA−: small for gestational age without postnatal catch up growth; SD: standard deviation; ns: not significant.

### MRI Data: Acquisition and Analysis

#### Mock Scanner Training Session

Movement artefacts are an important source of noise when acquiring MRI scans in young children. Therefore, children practiced the scanning session in a mock scanner under supervision of a pediatrician or a neuropsychologist to ensure acquaintance with the scanner. Such training has shown to be helpful and significantly reduces head movement [23]. The mock scanner closely resembled the MRI scanner used for the image acquisition, and was equipped with a manually operated patient table, head coil, foam cushions, headphones and earplugs. Speakers inside the bore reproduced the sounds of various scan sequences that can be heard during actual MRI investigations.

#### Data Acquisition

All data were collected at a 1.5-T Sonata scanner (Siemens, Erlangen, Germany) using an eight-channel phased-array head coil. A high-resolution T1-weighted scan using a 3D Magnetization Prepared Rapid Gradient Echo (MPRAGE) sequence was acquired in all children [24] (TR 2700 ms; TE 3.97 ms; voxel size 1.0*1.0*1.5 mm; flip angle 8°; 160 coronal slices; FOV of 250 mm covering whole brain; acquisition time 4.9 minutes).

#### MRI Data Analysis

MRI scans were visually inspected by a radiologist for structural abnormalities (FB). Subsequently, all T1 weighted scans were analysed using Freesurfer (version 4.2, http://surfer.nmr.mgh.harvard.edu/fswiki, Figure S1). In summary, this analysis included the reconstruction and parcellation of the cortical sheet of each hemisphere into 34 regions, used for the measurement of cerebral and cerebellar grey and white matter volume, cortical surface area and cortical thickness. For each individual dataset grey and white matter tissue and cerebrospinal fluid were classified, after intensity normalization and ‘skull stripping’. Next, using the grey/white matter segmentation, a surface tessellation was generated for the boundary between grey and white matter and for the boundary between grey matter and cerebrospinal fluid, for each hemisphere separately. Subsequently, cerebral cortical thickness of each point along the cortical mantle was computed by measuring the distance between the white and grey matter surface reconstructions. Automated parcellation of each individual cortical hemispheric sheet and subcortical structures resulted in the automatic segmentation of the cerebral and cerebellar cortex and subcortical structures (covering left and right hippocampus, amygdala, caudate nucleus, globus pallidum, putamen and thalamus). To ensure accurate automated segmentation using Freesurfer, each segmented brain was visually assessed by an experienced rater (MPvdH). Average volume of each of these parcellated brain regions and cerebral cortical surface were computed. Finally, cerebral and cerebellar grey and white matter volume and total cerebral volume were computed as the summation of the brain parameters of these regions. These steps are similar to the methods of Martinussen et al., examining T1 images of SGA+ adolescents, to increase comparability of our findings in young SGA children with brain development at 15 years of age [11].

We tested for differences between the SGA and AGA data by 1) comparison of global volumetric measures/cortical surface area and 2) regional comparison of point-specific differences in thickness values of the cortical mantle.

To investigate the influence of dexterity, the analysis was subsequently performed in the subgroup of right handed children only.

### Estimation of Intelligence Quotient

Prior to the MRI investigation (seven weeks to two days), children underwent neuropsychological examination. Intelligence quotient (IQ) was estimated on the basis of a four-subtest short form of the Wechsler's scales, yielding an estimate of the Full Scale IQ that would ordinarily be obtained by administration of the complete scales. Estimates of reliability and validity indicate the abbreviated forms of the Wechsler Preschool and Primary Scale Intelligence – Revised (WPPSI-R, Dutch version), for children under 6 years and the Wechsler Intelligence Scale for Children – third Edition (WISC-III, Dutch version) for children 6 years and older to approximate the Full Scale IQ when time limitations are a consideration [25]. Non-verbal IQ was estimated using Raven's Coloured Progressive Matrices in all children (CPM, Dutch version). Parental educational levels were assessed according to the International Standard Classification of Education 1997 [26].

### Statistical Analysis

Statistical analyses, other than those included in Freesurfer were performed using SPSS version 16.0 (Chicago, IL, USA). Analysis of baseline characteristics was performed using analysis of variance (ANOVA). Chi-square test was used for categorical baseline characteristics (sex, handedness and parental educational levels). P-values<0.05 were considered statistically significant.

Group comparison of global volumetric measures/cortical surface area (analysis part 1) and IQ was performed using a general linear model (GLM) analysis (multivariate) with subject group (AGA and SGA total (combined SGA− and SGA+), gender and lateralisation (left vs. right hemisphere) as fixed factors. Subsequently, to investigate a trend between the three subgroups (AGA vs. SGA+ vs. SGA−) a linear polynomial contrasts analysis was included in the GLM with subject group AGA vs. SGA+ vs. SGA− as fixed factor. Relationships between continuous data were assessed using Pearson's correlations. Age was used as a covariate in all analyses. P-values<0.05 (two tailed, Bonferroni-corrected for multiple comparisons) were considered statistically significant.

Regional comparison of point-specific differences in thickness values of the cortical mantle between AGA versus SGA and subsequent subgroup comparison (AGA versus SGA+ and AGA versus SGA−, SGA+ versus SGA−) was performed using GLM as implemented in Freesurfer, with age and gender as covariates. A threshold of p<0.001 (two-tailed uncorrected) was used. To examine whether effects survived correction for multiple testing, a cluster-wise correction for multiple comparisons was performed, as implemented in Freesurfer (Z-score: 2.33 (p<0.05), number of iterations: 5000).

### Ethics

The study was approved by the ethics committee of the VU University Medical Center, Amsterdam, The Netherlands. Written informed consent was obtained from the parents or guardians of each child and obtained according to the Declaration of Helsinki (BMJ 1991; 302: 1194).

## Results

The characteristics of the study groups AGA, SGA+ and SGA− are listed in Table 1. Groups were matched for age and gender. Forty-eight children were right handed and seven were left handed. The left handed children were equally distributed among the different subgroups. The gestational age did not differ between the groups. As expected, birth weight and head circumference at birth were lower in both SGA groups compared to AGA, although the brains were relatively spared. The SGA− and SGA+ group did not differ with respect to birth weight and head circumference at birth. Body length and head circumference at the time of MRI investigation was significantly lower in the SGA− group when compared with either the SGA+ (length SD: MD = −2.64, p<0.0001; head circumference SD MD = −1.30, p<0.0001) or AGA group (length SD: MD = −3.00, p<0.0001; head circumference SD MD = −1.79, p<0.0001).

One child had an isolated cerebellar cyst and was excluded for analysis of cerebellar measurements. There was no discernible delay in myelination in any child (as checked by an expert radiologist FB). There were no significant differences in volumes, cerebral cortical surface area or thickness between measures of the left or right hemisphere. Measures in both hemispheres are therefore reported without mutual comparison.

When comparing AGA children with the overall group of SGA children (Table 2), SGA children were found to have smaller total cerebral brain volume (p = 0.002) with smaller cortical surface area (right hemisphere: p<0.0001, left hemisphere: p<0.0001). The volume of the white matter of both cerebral (right hemisphere: p = 0.001, left hemisphere: p = 0.001) and cerebellar hemispheres (right hemisphere: p<0.0001, left hemisphere: p<0.0001) was significantly smaller in SGA children compared to AGA children. Furthermore, overall volumes of basal ganglia and thalamus were lower in the SGA group. Cortical grey matter volume of both the cerebrum (right hemisphere: p = 0.04, left hemisphere: p = 0.03). and the cerebellum (right hemisphere: p = 0.013, left hemisphere: p = 0.044) was lower in the SGA children but the effect on grey matter was less pronounced than on white matter. Only the right hippocampal volume was lower in SGA children (right hemisphere: p = 0.034, left hemisphere: p = 0.186) while there was no difference in the amygdala (right hemisphere: p = 0.161, left hemisphere: p = 0.311).

**Table 2:** Global cerebral and cerebellar measures in small for gestational age and appropriate for gestational age children (n = 55).

					Main effect AGA vs SGA		Main effect subgroup analysis (AGA vs SGA+ vs SGA−)		AGA vs SGA+	AGA vs SGA−	SGA+ vs SGA−	Polynomial trend analysis
	AGA	SGA			F-value	p-value						
		SGA+	SGA−	SGA total			F-value	p-value	p-value	p-value	p-value	p-value
Total cerebral volume in cm3	978.79 (99.58)	904.01 (77.49)	884.38 (106.45)	895.83 (89.78)	10.5	0.002	5.1	0.009	0.026	0.023	ns	0.008
Cerebral measures												
Cortical grey matter volume R in cm3	272 (24)	257 (27)	255 (28)	256 (27)	4.4	0.04	2.1	ns	ns	ns	ns	0.093
Cortical grey matter volume L in cm3	273 (26)	255 (24)	256 (29)	255 (26)	5.3	0.03	2.6	0.09	ns	ns	ns	ns
White matter volume R in cm3	206 (26)	188 (19)	179 (27)	184 (22)	12.6	0.001	6.5	0.003	0.02	0.005	ns	0.002
White matter volume L in cm3	206 (26)	186 (18)	179 (26)	183 (22)	13.4	0.001	6.7	0.003	0.012	0.006	ns	0.002
Cortical surface area R in cm2	883 (89)	795 (75)	783 (88)	789 (81)	18.0	<0.0001	8.9	0.001	0.001	0.004	ns	0.001
Cortical surface area L in cm2	880 (87)	787 (71)	780 (83)	783 (76)	21.2	<0.0001	10.5	<0.0001	<0.0001	0.002	ns	0.001
Caudate nucleus R in cm3	4.00 (0.47)	3.73 (0.36)	3.58 (0.53)	3.67 (0.44)	6.1	0.017	3.1	0.05	ns	0.069	ns	0.023
Caudate nucleus L in cm3	3.59 (0.46)	3.64 (0.44)	3.51 (0.48)	3.92 (0.34)	7.0	0.011	3.5	0.037	ns	0.049	ns	0.016
Globus pallidum R in cm3	1.81 (0.19)	1.67 (0.17)	1.48 (0.19)	1.59 (0.20)	15.3	<0.0001	12.7	<0.0001	0.05	<0.0001	0.017	<0.0001
Globus pallidum L in cm3	1.82 (0.31)	1.49 (0.27)	1.67 (0.16)	1.59 (0.23)	9.0	0.004	6.6	0.003	ns	0.002	ns	0.001
Putamen R in cm3	6.11 (0.63)	5.61 (0.63)	5.32 (0.65)	5.49 (0.65)	11.8	0.001	6.8	0.003	0.05	0.002	ns	0.001
Putamen L in cm3	6.37 (0.83)	5.97 (0.62)	5.59 (0.77)	5.81 (0.7)	6.7	0.012	4.4	0.017	ns	0.015	ns	0.005
Thalamus R in cm3	7.33 (0.69)	7.00 (0.48)	6.69 (0.77)	6.87 (0.63)	6.0	0.018	3.6	0.03	ns	0.032	ns	0.011
Thalamus L in cm3	7.45 (0.69)	7.10 (0.53)	6.67 (0.85)	6.90 (0.70)	7.3	0.009	4.5	0.015	ns	0.013	ns	0.004
Hippocampus R in cm3	4.06 (0.47)	3.91 (0.33)	3.72 (0.30)	3.83 (0.32)	4.7	0.034	3.5	0.04	ns	0.032	ns	0.011
Hippocampus L in cm3	3.83 (0.42)	3.72 (0.31)	3.62 (0.38)	3.68 (0.34)	1.8	ns	1.2	ns	ns	ns	ns	ns
Amygdala R in cm3	1.66 (0.26)	1.58 (0.24)	1.51 (0.21)	1.56 (0.23)	2.0	ns	1.1	ns	ns	ns	ns	ns
Amygdala L in cm3	1.66 (0.23)	1.60 (0.22)	1.57 (0.25)	1.59 (0.23)	1.1	ns	0.5	ns	ns	ns	ns	ns
Cerebellar measures*												
Grey matter volume R in cm3	54.90 (6.01)	53.00 (4.70)	48.77 (4.87)	51.21 (5.20)	6.7	0.013	7.2	0.002	ns	0.002	0.031	0.001
Grey matter volume L in cm3	54.34 (6.96)	53.36 (4.85)	48.45 (5.00)	51.25 (5.43)	4.3	0.044	6.7	0.003	ns	0.003	0.014	0.001
White matter volume R in cm3	11.77 (1.85)	10.27 (0.92)	9.48 (1.59)	9.93 (1.29)	22.0	<0.0001	12.0	<0.0001	0.003	<0.0001	ns	<0.0001
White matter volume L in cm3	11.60 (1.95)	10.34 (0.95)	9.40 (1.49)	9.93 (1.28)	17.6	<0.0001	10.6	<0.0001	0.012	<0.0001	ns	<0.0001

Data are presented as mean (± standard deviation); p-value<0.05 is considered significant, p-values between 0.05 and 0.1 are reported;

*: results of cerebellar measures should be interpreted with caution due to restricted resolution of MRI settings.

Abbreviations: AGA: appropriate for gestational age; SGA+ small for gestational age with postnatal catch up growth; SGA−: small for gestational age without postnatal catch up growth; R: right hemisphere; L: left hemisphere; ns: not significant.

**Figure 1:**
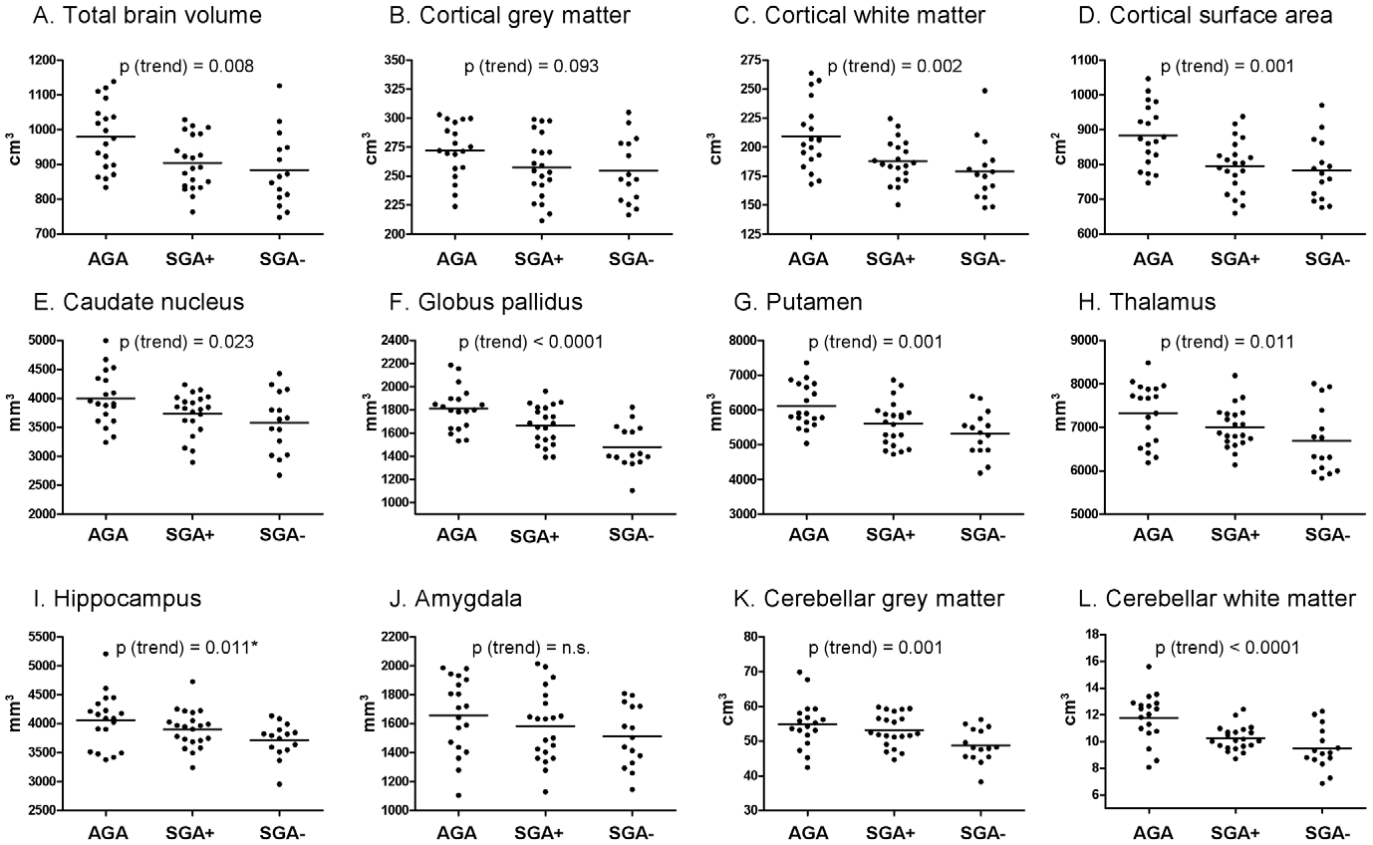
Global brain parameters in AGA, SGA+ and SGA− children. Only data from the right hemisphere are shown. Bars depict the mean within each group. P-values for trend derived from polynomial contrast analyses are shown (see also Table 2). P-values of the left hemisphere are mentioned only when statistically different compared to p-values of the right hemisphere. AGA: appropriate for gestational age; SGA+: small for gestational age with catch-up growth; SGA−: small for gestational age without catch-up growth; n.s.: not signifcant. *: P-value of left hemisphere not significant.

To investigate the effect of catch-up growth on the brain, we subsequently investigated the relation between AGA, SGA+ and SGA− children. Subgroup analysis showed that the largest difference was observed between the AGA and SGA− groups (Table 2) and that the SGA+ subgroup constituted an intermediate between the other groups (Table 2, Figure 1). Differences between the AGA, SGA+ and SGA− groups were present in both boys and girls. Total cerebral volume, cerebral white matter volume, cortical surface area, basal ganglia and cerebellar volume all showed a significant decrease from AGA to SGA+ to SGA− (Figure 1). For cerebral cortical grey matter, the hippocampus and amygdala, a similar trend was observed (Figure 1); however, the trend did only reach significance for the hippocampus of the right hemisphere (Table 2).

**Figure 2:**
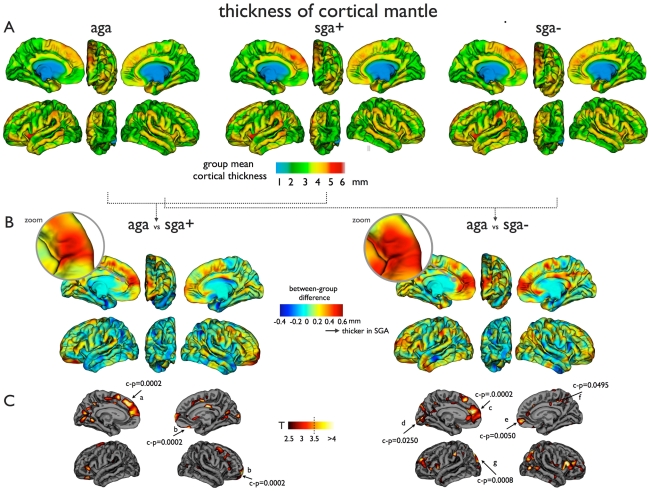
Cortical thickness of the cerebral cortical mantle in AGA, SGA+ and SGA− children. Figure shows significant cortical thickening in children born SGA in comparison to normal AGA children. Specifically, most pronounced thickening is found in frontal brain regions, overlapping medial frontal and superior frontal cortices. Figure 2A shows the cortical thickness of the cerebral cortical mantle in the AGA, SGA+ and SGA− group, respectively. Figure 2B shows the effect-size difference maps between the AGA and SGA+ and AGA and SGA− group, showing strong thickening of the medial frontal and superior frontal regions in both SGA children. Figure 2C shows the statisical difference maps between AGA vs SGA+ and AGA vs SGA− , thresholded at p<0.001. Both SGA+ and SGA− children showed wide-spread signifiant higher thickness of the cortical mantle, most pronounced in frontal (as marked as the frontal cluster) and parietal regions, surviving cluster-wise correction for multiple testing (see materials and methods). Regions a–g refer to regions in Table 3 (AGA vs SGA+: a = Superior frontal, b = Lateral orbitofrontal; AGA vs SGA−: c = Superior frontal, d = Pericalcarine, e = Superior frontal, f = Posterior cingulated, g = Superior parietal). AGA: appropriate for gestational age; SGA+: small for gestational age with catch-up growth; SGA−: small for gestational age without catch-up growth; c–p: p-value after cluster-wise correction for multiple comparisons.

**Figure 3:**
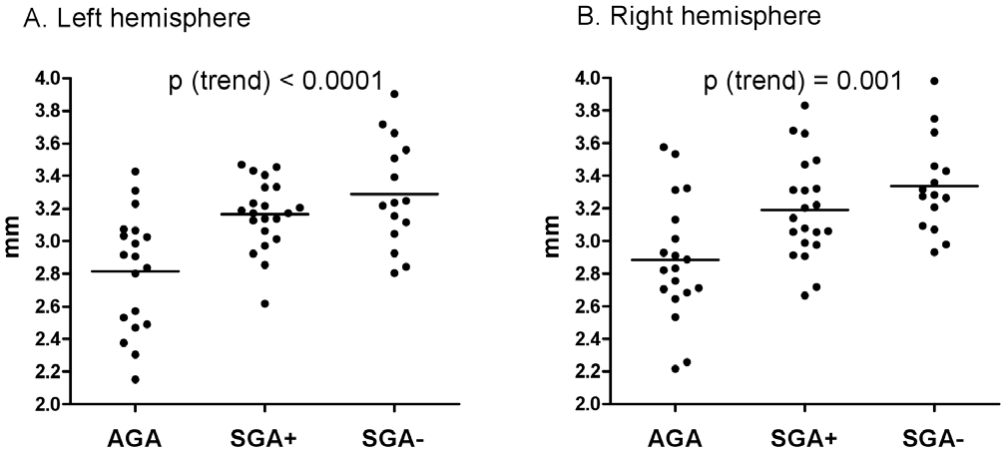
Cortical thickness of the medial prefrontal cortex of both hemispheres in AGA, SGA+ and SGA− children. P-values for trend derived from polynomial contrast analyses are shown. Bars depict the mean within each group. AGA: appropriate for gestational age; SGA+: small for gestational age with catch-up growth; SGA−: small for gestational age without catch-up growth.

For the second part of our analysis we performed a regional comparison of cortical thickness of the cerebral cortical mantle (Figure 2A). Comparing AGA and SGA children, distinct areas of mainly thicker cortex were present in the SGA groups (Figure 2B). Local thickening was most pronounced in a substantial part of the frontal lobe of both hemispheres (superior and medial frontal cortex, P<0.001 two tailed). In addition, a thicker cortex was found in discrete regions of the posterior cingulate cortex, lateral orbitofrontal gyrus, angular gyrus and the pericalcarine region in SGA− and SGA+ children. Small areas of thinner cortex were also present and were located in the middle temporal gyrus and subcentral area. Statistical difference maps demonstrated that the most pronounced difference in cortical thickness was found in the frontal regions (Figure 2C), which remained significant after cluster-wise correction for multiple comparisons (cluster-wise p<0.05, see method section. For other regions see Table 3). Both SGA+ and SGA− displayed a thicker superior and medial frontal cortex compared to AGA. The frontal ROI was selected to further examine the cortical thickness of the frontal cortex between AGA and SGA children. For this, the ROI was selected as those vertices that showed a significant difference between AGA and SGA, showing a T>3.5. Similar to the global brain parameters, the cortical thickness of the frontal cluster displayed a significant trend between the subgroups (from AGA to SGA+ to SGA−, Figure 3), with SGA− children having the thickest cortex.

**Table 3:** Regions of thicker cortex, significant after cluster-wise correction for multiple comparisons (P<0.05), in SGA+ and SGA− children compared to AGA children (n = 55 children).

Region*	Anatomical region (Talairach coordinates)	x	y	z
AGA vs SGA+				
a	Superior frontal	−8,2	45,4	34,9
b	Lateral orbitofrontal	11,2	48,6	−18,8
AGA vs SGA−				
c	Superior frontal	−9,9	50,6	14,8
d	Pericalcarine	−11,8	−84,7	2,0
e	Superior frontal	8,7	62,4	5,0
f	Posterior cingulate	6,8	−12,3	29,8
g	Superior parietal	−20,5	−76,1	42,0

Cortical areas showing significant group interactions (cluster-wise correction for multiple comparisons p<0.05) between AGA vs SGA+ and AGA vs SGA−.

*Regions refer to regions in Figure 2 C.

Abbreviations: AGA: appropriate for gestational age; SGA+ small for gestational age with postnatal catch up growth; SGA−: small for gestational age without postnatal catch up growth.

The subgroup of right handed children did not significantly differ compared to the total study population with respect to baseline characteristics. Also for the right-handed children, we found similar differences between AGA, and SGA children for both the global brain measures as well as the regional comparison of cortical thickness of the cerebral cortical mantle. (Tables S1, S2, S3, Figures S2, S3, S4). In general, effects on cerebellar and cerebral cortical grey and white matter volumes were more pronounced in the subgroup of right-handed children (higher F-values). Regional comparison of the cortical thickness of the cortical mantle demonstrated that after cluster-wise correction for multiple comparisons, only the clusters for the SGA− vs AGA group remained significant.

IQs of the children were estimated with Wechsler scales and Raven's CPM. In the total sample, the difference between IQs estimated with the two different methods was only 0.2 IQ-points (mean Wechsler Scales IQ = 107.7±13.5 vs mean CPM-IQ = 107.9±13.7). Furthermore, no significant difference existed between Wechsler scales IQ and CPM-IQ in either subgroup. Therefore, only Wechsler scales IQ are reported. SGA children showed lower IQ scores compared to AGA children (mean IQ SGA: 104.3; SD: 11.3 vs. mean IQ AGA: 113.1; SD 15.7; F 7.2, P = 0.010). Subgroup comparison showed that the IQ of children in the SGA+ group was higher than of children in the SGA− group (mean IQ SGA+ 106.8; SD 11.4 vs. mean IQ SGA− 100.9; SD 10.6) but this difference did not reach significance (p = 0.526). Linear polynomial contrast analysis showed a significant trend for IQ between the three groups (F: 9.048, p = 0.004). We did not observe significant correlations between IQ and brain measures in any of the subgroups. Importantly, in the AGA subgroup, children with IQ>110 had brain measures in similar range and did not differ significantly from AGA children with IQ≤110.

The proportion of parents in the SGA group with an educational level confined to first stage of basic education or lower secondary education was higher compared to the parents in the AGA group (mothers SGA 17.6% vs AGA 0%, (Fisher exact p = 0.16, two tailed; fathers SGA 20.6% vs AGA 5.6%, Fisher exact p = 0.24, two tailed). We did not observe a difference in level of education between parents of SGA+ children compared to SGA− children.

## Discussion

The results of the current study demonstrate that being born small for gestational age (SGA) is associated with altered anatomy of the brain at the age of four to seven years. SGA children showed reduced cerebral and cerebellar white matter volumes, smaller volumes of basal ganglia together with a smaller overall cortical surface area. SGA children showed a regionally thicker cortical layering, most pronounced in the medial and superior frontal cortex. Differences were present in the total sample and the subgroup of right handed children only. Although differences in brain structure were most pronounced in SGA children without postnatal catch-up growth (SGA−), our results demonstrate that postnatal catch-up growth (SGA+) of the body does not result in full recovery of brain volume and morphology.

We examined the anatomy of cortical, cerebellar and sub-cortical structures in 4–7 year old SGA born children, as well as the thickness and surface area of the cortical mantle using a surface-based analytic approach. Direct measurement of the cortical mantle reduces the risk of partial volume effects, which may be a benefit relative to other techniques such as for instance voxel based morphometry, and allows for detecting more subtle focal cortical differences [27], [28]. To our knowledge, validation studies concerning cortical thickness and cortical surface in children do not exist but surface based cortical thickness measurements have already been used in other pediatric populations [29]–[31]. Tissue classification, a fundamental step in cortical thickness measurement, is known to be especially difficult in young pediatric populations (infants and toddlers), due to low contrast between grey and white matter [32]. As mentioned, in our study, we verified that there were no children with a discernable delay in myelination and each segmented brain was visually assessed for accuracy.

This study has a cross-sectional design. Future MRI studies with a longitudinal design, preferentially from gestation onwards, are required to document brain development in SGA populations investigating whether anatomical differences are present already at birth and whether these differences persist, become more pronounced or gradually disappear with age. Because being born SGA is associated with impairments in several cognitive domains, the primary focus of the current study was to investigate the anatomy of the cerebral cortex. However, it is of high interest to examine other aspects of brain anatomy and development in SGA children as well. Indeed, our results show a robust decrease in white matter volume, suggesting that, besides the altered structure of cortical and sub-cortical grey matter regions, the integrity of white matter connections between these regions may also be affected in SGA children. Studies have demonstrated that the development of brain connectivity is vital for healthy cognitive functioning [33], [34] suggesting that future studies examining connectivity aspects of both grey and white matter in SGA children using modern imaging techniques like functional MRI and Diffusion Tensor Imaging are of high importance. Finally, animal studies would allow histopathological examination of brain tissue and will improve our knowledge about brain anatomy and development following intrauterine growth restriction.

Interestingly, the observed differences in brain anatomy in SGA children corroborate results of animal studies. Sheep and rodent animal models for SGA demonstrate a reduced brain weight, together with reduced white matter volume with delayed and reduced myelination of both cerebrum and cerebellum in newborn offspring, which overlaps our current observation in SGA children. [35]–[37]. In addition, reduced volumes of cerebral and cerebellar cortical grey matter with a reduced number of neurons, reduced cell size, compromised synaptogenesis and delayed neuronal migration have been reported in rats [7]. Studies in SGA animal models investigating the cortical mantle for regional differences are lacking, however. Furthermore, reduced volumes of basal ganglia and hippocampal volumes have been described in newborn guinea pigs [5], [38]. A more recent animal study investigated whether the effects of adverse prenatal conditions on brain structure persist into adulthood and demonstrated that alterations in brain structure were still present in adult guinea pigs [10]. The nature and extent of the neuropathology varies in different SGA models and is related to the severity of the insult and the timing of the insult in relation to the gestational age [6], [37], [39], [40]. Therefore, care needs to be taken when comparing results of SGA animal models to human studies.

Comparison of our data with other human MRI studies is hampered by the fact that the study cohorts differ considerably. For instance, some studies have been performed in very prematurely born SGA children [14], [41], [42]. Because prematurity, besides being born SGA, is known to affect brain structure, it is difficult to compare the results of the current study with the results of these prematurely born study cohorts [43], [44]. Only one research group has also scanned children who were born SGA at term [11], [12]. In these studies, SGA adolescents with catch-up growth were scanned at the age of 15 years. The results were in line with the results of our study, as SGA adolescents were found to have smaller brains with reduced white matter volume. There were no significant reductions in cerebral cortical grey matter volume or hippocampal and amygdala volume. Moreover, a similar thickening of the cortex in the frontal lobe was described [11]. The authors speculated that a delay in cortical maturation in SGA adolescents compared to healthy controls is responsible for the thicker cortex in SGA individuals. However, normal cortical development is characterized by an initial increase in cortical thickness during childhood followed by progressive thinning in adolescence [45], [46]. When we combine the results of the study of Martinussen et al. with the results of the current study, demonstrating that the thicker cortex is already present in young 4–7 years old SGA children, we hypothesize that an altered rather than a delayed maturation results in a different layering of the cortical mantle in SGA individuals.

Children born SGA have suffered from an adverse intrauterine environment leading to intrauterine growth restriction. The most common cause of intrauterine growth restriction is placental insufficiency during the second half of pregnancy [47]. It seems therefore plausible that differences in brain anatomy between SGA children and normal children are related to developmental events that take place during the second half of pregnancy. Normal brain development in this time frame involves both development of grey and white matter [48], [49]. In grey matter, elaboration of dendritic and axonal ramifications, establishment of synapses and programmed cell death of neuronal processes and synaptic pruning takes place. It is unlikely that neurogenesis and neuronal migration are involved because the peak time period for these processes is during the first half of pregnancy. Proliferation and differentiation of glia advances and gyral formation starts during second half of pregnancy. Formation of white matter starts in the second half of pregnancy and begins with an increase in the number of oligodendrocytes. Myelination continues throughout pregnancy and peaks after birth. The observed reduction in white matter may result from a reduced number of oligodendrocytes or a reduced capacity of these oligodendrocytes to form myelin, or both [37]. These may lead to thinner sheaths of myelin that affect axonal conduction velocity, and may contribute to impaired neuronal function. Interestingly, SGA children showed a much more pronounced reduction in cortical white than in grey matter volume. The greater reduction in cortical white matter in SGA children may reflect a relative sparing of grey matter during the second half of pregnancy, or differential compensatory growth during early postnatal life.

Our findings of focal thickening of the cortex in SGA children may be explained by a similar mechanism, i.e. that various organizational events are differentially compromised. For instance, a reduced apoptosis and synaptic pruning or compromised intracortical myelination [6], [40] may result in regional cortical thickening. An alternative mechanism related to focal thickening is a reduced cortical gyrification and sulcation rather than primary abnormalities in the cortex itself [39], [42], [48]. Indeed, our findings show a reduced cortical surface area without a significantly lower cortical volume in brains of SGA children, suggesting a diminished folding pattern in SGA children. Future studies using animal models for SGA are needed to investigate changes of the cortex, especially frontal, at the microscopic level.

Detailed consideration of the anatomical differences of specific regions between AGA and SGA children and their possible relationship with differences in cognitive development is beyond the scope of this study but some suggestions for future studies can be made. Most pronounced differences in cortical thickness were found in medial prefrontal areas involved in executive function and decision making and this will be a focus for neuropsychological testing. Moreover, the superior parietal cortex and posterior cingulate cortex are part of the default mode network which is another focus for future study.

A key finding of our study is that postnatal bodily catch-up growth with normalization of head circumference in SGA children does not necessarily imply complete normalization of brain morphology. Differences in brain anatomy in SGA children were most pronounced in SGA− children; SGA+ children constitute an intermediate group between the SGA− and AGA group. This indicates that careful observation of the SGA+ subgroup is warranted, as cognitive impairments may be missed because bodily growth catch-up has occurred. Moreover, as our findings suggest, the SGA− subgroup had even more pronounced differences in brain anatomy, with more greatly reduced white matter volume and increased cortical thickening in the medial frontal cortex. These findings may fit long-term follow up neuropsychological studies in SGA children with and without catch-up growth, reporting a similar trend with the most severe cognitive limitations in the group of patients without postnatal catch-up growth (i.e. similar to our SGA− group) [9], [17], [21], [50], [51].

In our study group, the SGA+ children did not only display catch-up growth of bodily parameters but also, partial catch-up growth of the brain. The only known postnatal determinant of catch-up growth in SGA children in developed countries is breast feeding which is associated with better outcome [52], [53]. In general, low socioeconomic status is associated with low birth weight, poor postnatal growth and lower levels of cognitive performance [54], [55]. The educational level of the AGA parents was higher. These effects are mediated by, among others, cognitive stimulation and nutrition [54], [56], [57]. To our knowledge, there are no clear data on the relation between socioeconomic status and catch-up growth in SGA children in developed countries. Interestingly, in the current study group, a higher educational level of the parents was not associated with catch-up growth in SGA children.

A limitation of the current study is the composition of the control group. This group was matched based on age and gender. However, the mean IQ of the AGA children was almost 1SD above population mean. We failed to observe associations between IQ and brain morphology within each subgroup [58], [59]. In contrast, a previous study performed in children reported that children with superior IQ had a thinner cortex before the age of eight years [58], a pattern which reversed in the second decade. The current study was not powered to address this question and our group size is probably too small to detect these relations. To exclude the possibility that most of the differences between AGA and SGA children are in fact caused by differences in IQ, we compared brain measures between low and high IQ groups within each subgroup and focused on the AGA group. None of the brain measures differed significantly between the low and high IQ group. Although the group size may be too low to allow any definite conclusion we think that the observed differences between the AGA and SGA groups are not explained solely by the high IQ levels of the AGA group.

In conclusion, our findings show that SGA children have smaller cerebral volumes coupled with a smaller cortical surface area. Furthermore, there is a widespread reduction in white matter volume of both the cerebral and cerebellar hemispheres. Regional differences in thickness of the cortical mantle are indicative of a different cytoarchitecture of the cerebral cortex in SGA children. Our findings show that postnatal catch-up growth of the body and head circumference (SGA+) does not result in normalization of brain morphology. The brains and IQ of SGA+ children are an intermediate between SGA− and AGA and children, indicating that careful follow-up of these children during school age is warranted. Longitudinal imaging studies need to reveal the course of neuroanatomical development in SGA children.

## Supporting Information

Figure S1 **Cortical segmentation Freesurfer.** Axial slices displaying tissue classification using Freesurfer. For each individual dataset grey and white matter tissue and cerebrospinal fluid were classified. Next, automated parcellation of each individual cortical hemispheric sheet and subcortical structures resulted in the automatic segmentation of the cerebral and cerebellar cortex and subcortical structures. Each automated segmented brain was visually checked for accuracy. Figure shows (as an example) the segmentation of the left cortical sheet (white = light green, grey matter = dark green), cerebellum, subcortical structures (see main text for included structures).
(TIF)

Figure S2 **Global brain parameters in right handed AGA, SGA+ and SGA− children.** Only data from the right hemisphere are shown. Bars depict the mean within each group. P-values for trend derived from polynomial contrast analyses are shown (see also Table 2). P-values of the left hemisphere are mentioned only when statistically different compared to p-values of the right hemisphere. AGA: appropriate for gestational age; SGA+: small for gestational age with catch-up growth; SGA−: small for gestational age without catch-up growth; n.s.:not signifcant. *: P-value of left hemisphere not significant.
(TIF)

Figure S3 **Cortical thickness of the cerebral cortical mantle in right handed AGA, SGA+ and SGA− children.** Figure shows significant cortical thickening in children born SGA in comparison to normal AGA children. Specifically, most pronounced thickening is found in frontal brain regions, overlapping medial frontal and superior frontal cortices. Figure 3A shows the cortical thickness of the cerebral cortical mantle in the AGA, SGA+ and SGA− group, respectively. Figure 3B shows the effect-size difference maps between the AGA and SGA+ and AGA and SGA− group, showing strong thickening of the medial frontal and superior frontal regions in both SGA children. Figure 3C shows the statisical difference maps between AGA vs SGA+ and AGA vs SGA− , thresholded at p<0.001. Both SGA+ and SGA− children showed wide-spread signifiant higher thickness of the cortical mantle, most pronounced in frontal (as marked as the frontal cluster) and parietal regions, surviving cluster-wise correction for multiple testing (see materials and methods). Regions a–g refer to regions in Table S3 (AGA vs SGA−: a = Superior frontal, b = Superior frontal, c = Superior frontal). AGA: appropriate for gestational age; SGA+: small for gestational age with catch-up growth; SGA−: small for gestational age without catch-up growth; c-p: p-value after cluster-wise correction for multiple comparisons.
(TIF)

Figure S4 **Cortical thickness of the medial prefrontal cortex of both hemispheres in right handed AGA, SGA+ and SGA− children.** P-values for trend derived from polynomial contrast analyses are shown. Bars depict the mean within each group. AGA: appropriate for gestational age; SGA+: small for gestational age with catch-up growth; SGA−: small for gestational age without catch-up growth.
(TIF)

Table S1 **Characteristics of subgroup of right handed children (n = 48).** Data (except gender and handedness) are presented as mean (± standard deviation); p-value<0.05 is considered significant, p-values between 0.05 and 0.10 are reported. *weight for length SD. Abbreviations: AGA: appropriate for gestational age; SGA+ small for gestational age with postnatal catch up growth; SGA−: small for gestational age without postnatal catch up growth; SD: standard deviation; ns: not significant.
(XLS)

Table S2 **Global cerebral and cerebellar measures in right handed small for gestational age and appropriate for gestational age children (n = 48).** Data are presented as mean (± standard deviation); p-value<0.05 is considered significant, p-values between 0.05 and 0.1 are reported; *: results of cerebellar measures should be interpreted with caution due to restricted resolution of MRI settings. Abbreviations: AGA: appropriate for gestational age; SGA+ small for gestational age with postnatal catch up growth; SGA−: small for gestational age without postnatal catch up growth; R: right hemisphere; L:left hemisphere; ns: not significant.
(XLS)

Table S3 **Regions with thicker cortex (Talairach coordinates) in SGA− children compared to AGA children (righthanded children, n = 48).** Cortical areas showing significant group interactions (cluster-wise correction for multiple comparisons thresholded at p<0.05) between AGA vs SGA− (AGA vs SGA+ not significant). * Regions refer to regions in Figure 2C. Abbreviations: AGA: appropriate for gestational age; SGA+ small for gestational age with postnatal catch up growth; SGA−: small for gestational age without postnatal catch up growth.
(XLS)
