# Supplementary figures and images for: Global and Regional Differences in Brain Anatomy of Young Children Born Small for Gestational Age
Source: PLoS One. 2011 Sep 13;6(9):e24116. doi: 10.1371/journal.pone.0024116 (PMC3172224; doi:10.1371/journal.pone.0024116)

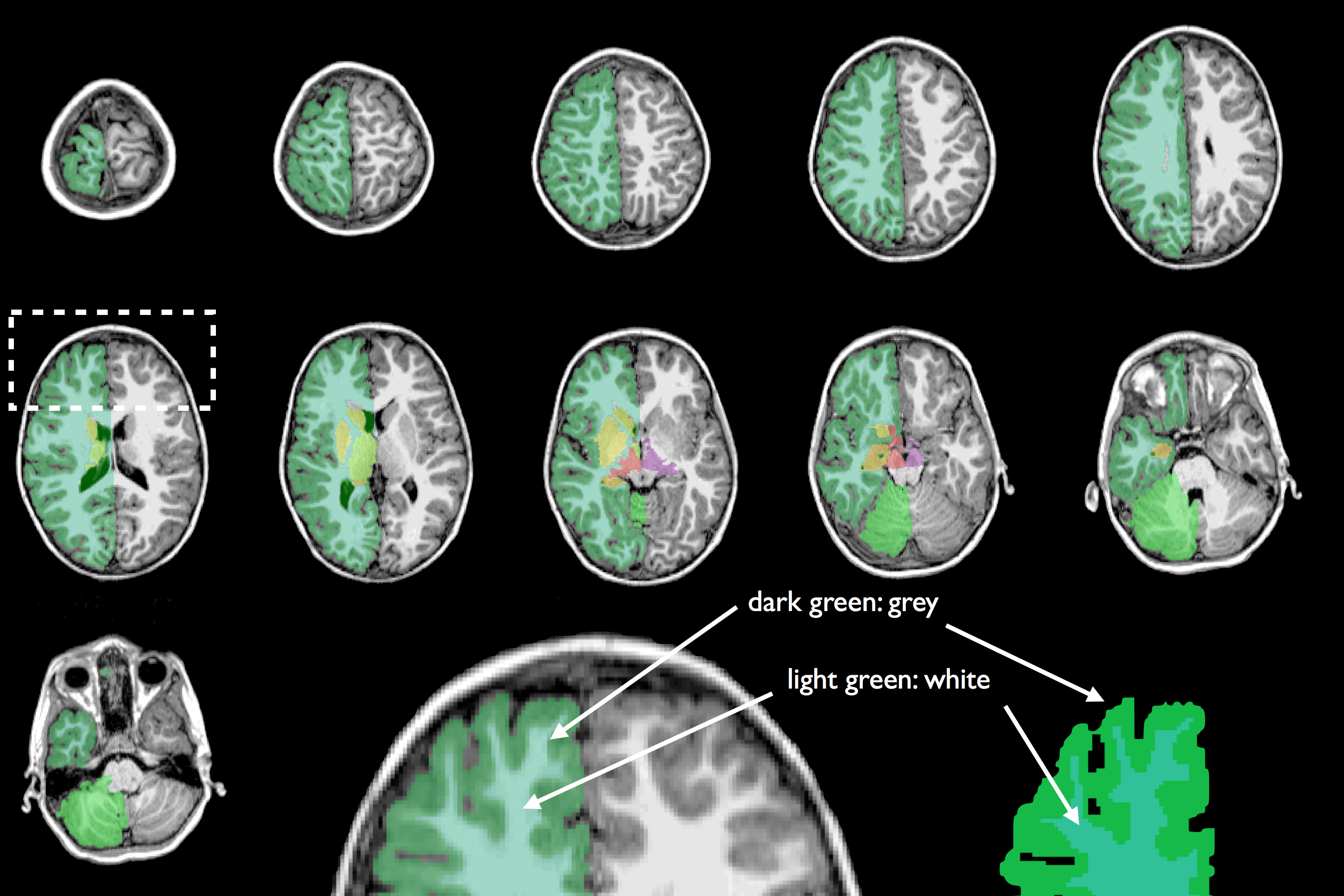

Supplement: Figure S1 — Cortical segmentation Freesurfer. Axial slices displaying tissue classification using Freesurfer. For each individual dataset grey and white matter tissue and cerebrospinal fluid were classified. Next, automated parcellation of each individual cortical hemispheric sheet and subcortical structures resulted in the automatic segmentation of the cerebral and cerebellar cortex and subcortical structures. Each automated segmented brain was visually checked for accuracy. Figure shows (as an example) the segmentation of the left cortical sheet (white = light green, grey matter = dark green), cerebellum, subcortical structures (see main text for included structures). (TIF) [file pone.0024116.s001.tif]

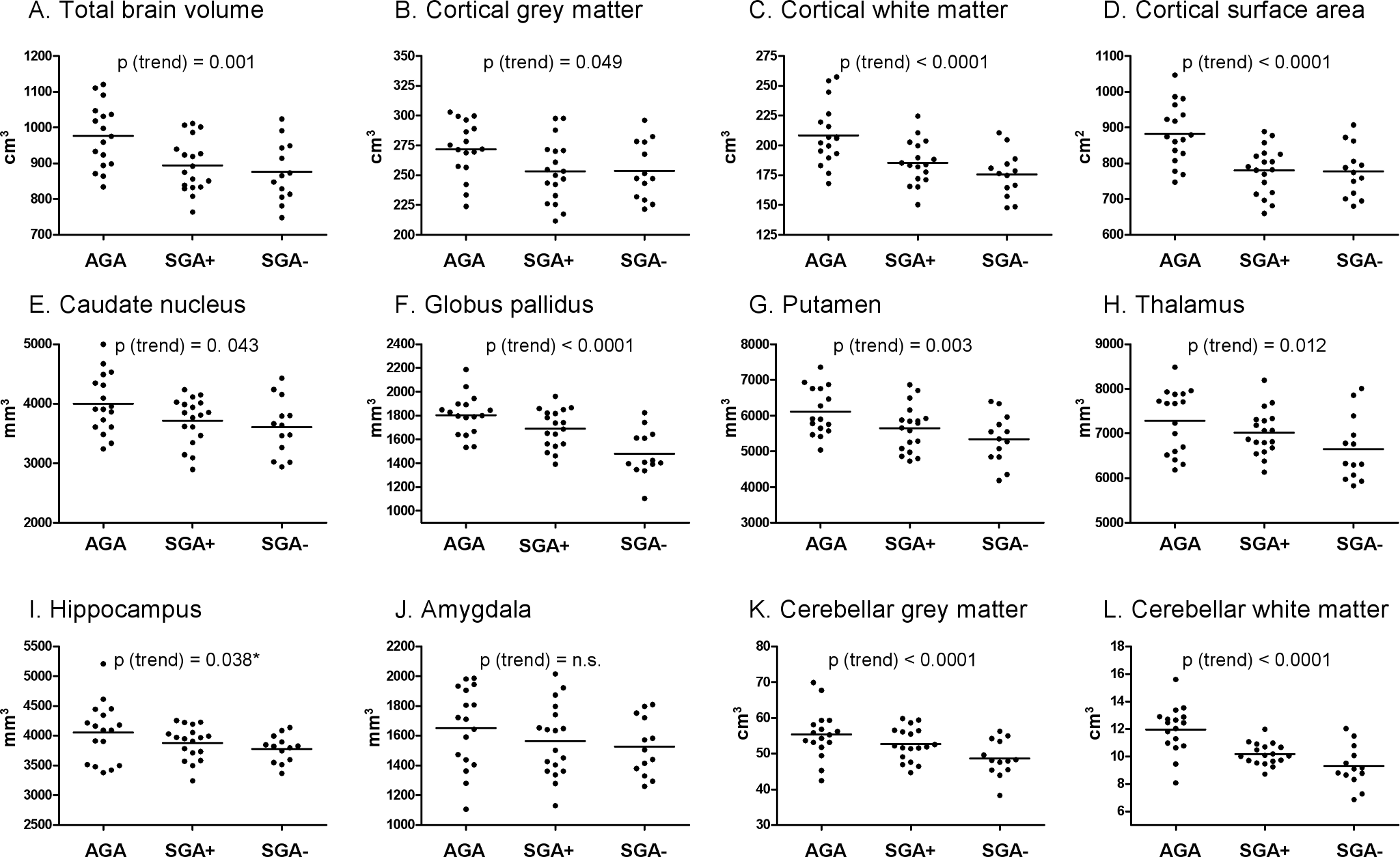

Supplement: Figure S2 — Global brain parameters in right handed AGA, SGA+ and SGA− children. Only data from the right hemisphere are shown. Bars depict the mean within each group. P-values for trend derived from polynomial contrast analyses are shown (see also Table 2). P-values of the left hemisphere are mentioned only when statistically different compared to p-values of the right hemisphere. AGA: appropriate for gestational age; SGA+: small for gestational age with catch-up growth; SGA−: small for gestational age without catch-up growth; n.s.:not signifcant. *: P-value of left hemisphere not significant. (TIF) [file pone.0024116.s002.tif]

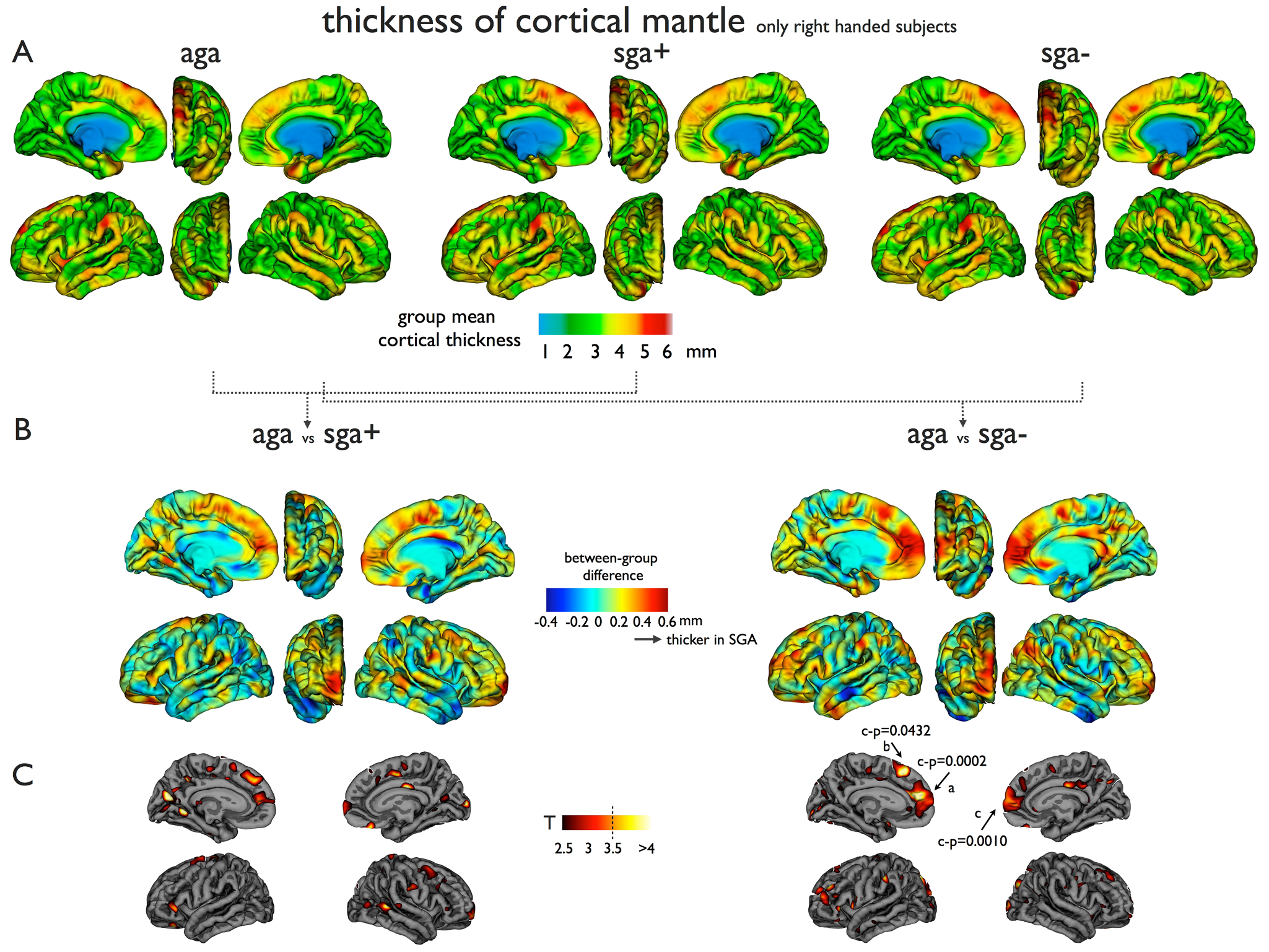

Supplement: Figure S3 — Cortical thickness of the cerebral cortical mantle in right handed AGA, SGA+ and SGA− children. Figure shows significant cortical thickening in children born SGA in comparison to normal AGA children. Specifically, most pronounced thickening is found in frontal brain regions, overlapping medial frontal and superior frontal cortices. Figure 3A shows the cortical thickness of the cerebral cortical mantle in the AGA, SGA+ and SGA− group, respectively. Figure 3B shows the effect-size difference maps between the AGA and SGA+ and AGA and SGA− group, showing strong thickening of the medial frontal and superior frontal regions in both SGA children. Figure 3C shows the statisical difference maps between AGA vs SGA+ and AGA vs SGA− , thresholded at p<0.001. Both SGA+ and SGA− children showed wide-spread signifiant higher thickness of the cortical mantle, most pronounced in frontal (as marked as the frontal cluster) and parietal regions, surviving cluster-wise correction for multiple testing (see materials and methods). Regions a–g refer to regions in Table S3 (AGA vs SGA−: a = Superior frontal, b = Superior frontal, c = Superior frontal). AGA: appropriate for gestational age; SGA+: small for gestational age with catch-up growth; SGA−: small for gestational age without catch-up growth; c-p: p-value after cluster-wise correction for multiple comparisons. (TIF) [file pone.0024116.s003.tif]

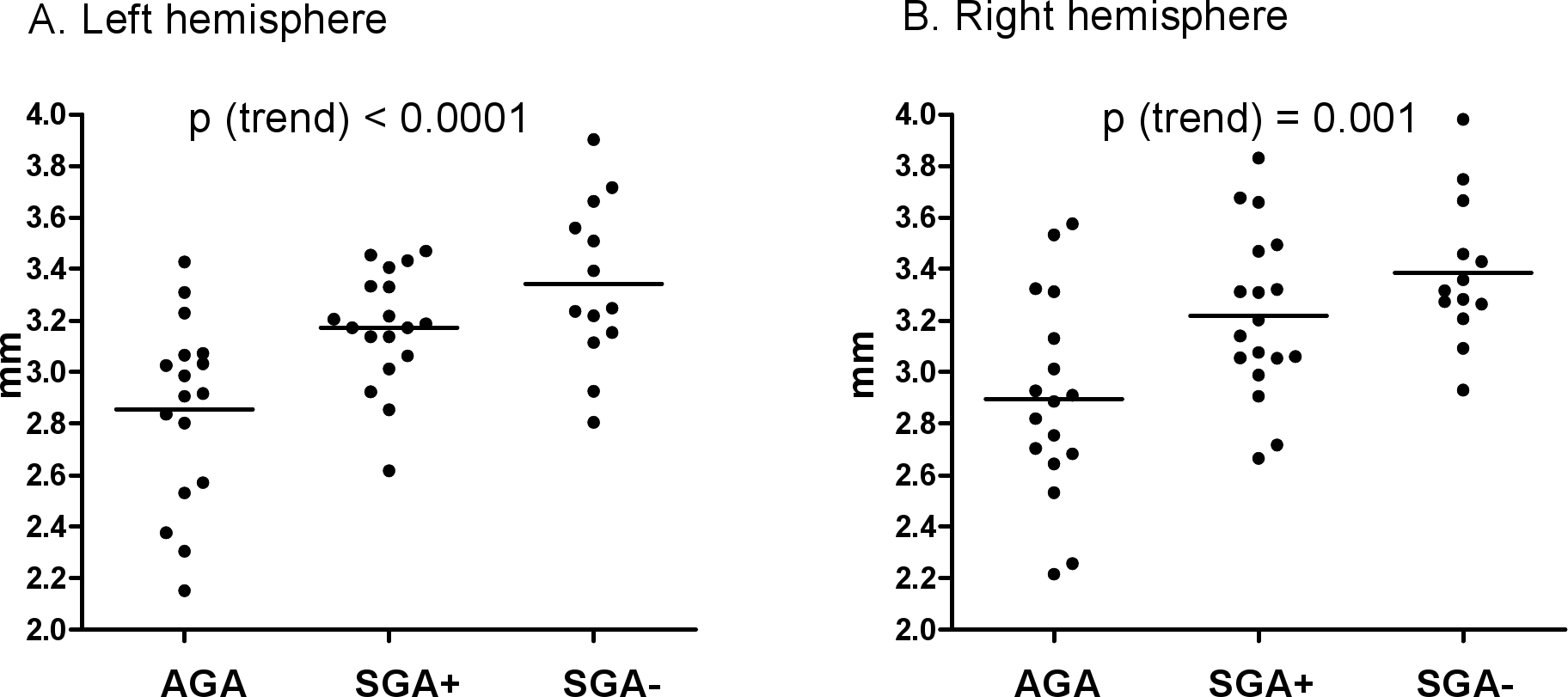

Supplement: Figure S4 — Cortical thickness of the medial prefrontal cortex of both hemispheres in right handed AGA, SGA+ and SGA− children. P-values for trend derived from polynomial contrast analyses are shown. Bars depict the mean within each group. AGA: appropriate for gestational age; SGA+: small for gestational age with catch-up growth; SGA−: small for gestational age without catch-up growth. (TIF) [file pone.0024116.s004.tif]
